# Emergency room advanced life support after cardiac arrest: outcomes and survival, nursing care and team response

**DOI:** 10.1186/cc13675

**Published:** 2014-03-17

**Authors:** A Villamor, A Tugas, Y Masguret, M Bayon, P Ceregido

**Affiliations:** 1 Barcelona Clinic Hospital, Barcelona, Spain

## Introduction

An organized team response and trained nursing staff in the emergency room critical patient area (ER-CPA) are the main factors to determine cardiopulmonary resuscitation (CPR) success rates. The objective was to evaluate outcomes in the emergency room after advanced life support (ALS) by reanimation teams, to improve nursing care, quality of resuscitation and survival.

## Methods

We included all adult patients receiving ALS in the ER-CPA during 1 year. Cases not allowed CPR are excluded. A retrospective design, multivariate with ALS performed, gender, age, cause of arrest, precedence, outcome and hospital derivation, and 100% of cases studied.

## Results

A total of 149 patients were attended in the ER-CPA with ALS maneuvers during the studied period (9.23% of a total 1,613). Thirty-four of them were nonheart-beating donors. The protocol was performed with 23 effective donors and 31 organs were obtained for transplant. Respiratory arrest (*n *= 31) was the best outcome group, with 100% survival and in-hospital ICU transfer. The cardiac arrest (CA) group depended on cause of CA: cardiac arrhythmia (*n *= 12), survival 50%; surgical etiology (nontraumatic), survival 50%, mean age 70; myocardial infarction (*n *= 12), survival 83%, mean age 61; sepsis (*n *= 6), survival 67%, mean age 48; drugs overdose (*n *= 2), survival 100%; traumatic cardiac arrest (*n *= 15), 89% mortality, mean age 41; and CA with unknown etiology, survival 38%, mean age 68.

## Conclusion

Emergency room cardiac arrest arrivals are not correlated with ER-CPA total activity. Good survival rates are probably related to a special quick response protocol and trained teams. Outcomes research to detect potential improvement in nurse care is needed, and should be evidence guided. Worst results in survival rates must be detected as the main topics for nursing care development.

